# AAPM WGEPA Report 437: An introduction to entrustable professional activities for medical physics residency education

**DOI:** 10.1002/acm2.70198

**Published:** 2025-10-15

**Authors:** Christina L. Brunnquell, Hania A. Al‐Hallaq, Derek W. Brown, Jay W. Burmeister, Kristi R. G. Hendrickson, John R Vetter, Laura Padilla

**Affiliations:** ^1^ University of Minnesota Department of Radiology Minneapolis Minnesota USA; ^2^ Department of Radiation Oncology Emory University Atlanta Georgia USA; ^3^ Department of Radiation Medicine and Applied Science University of California, San Diego La Jolla California USA; ^4^ Karmanos Cancer Center Wayne State University School of Medicine Detroit Michigan USA; ^5^ Department of Radiation Oncology University of Washington Seattle Washington USA; ^6^ Department of Medical Physics University of Wisconsin Madison Wisconsin USA

**Keywords:** clinical training, competency‐based medical education, entrustable professional activities, medical physics residency

## Abstract

Most modern medical physics residency programs consist of a 24‐month clinical training curriculum based on standards and recommendations of medical physics organizations such as the Commission on Accreditation of Medical Physics Education Programs (CAMPEP) and American Association of Physicists in Medicine (AAPM). Although these recommendations are extensive, their implementation is inconsistent across programs, and the quality of resident evaluation and feedback is highly variable. Competency‐based medical education (CBME) is a learner‐centered educational approach that focuses on whether the learner is acquiring the knowledge, skills, and attitudes required to become a competent professional upon graduation. Entrustable professional activities (EPAs) are an example of a CBME approach that could improve the preparation of medical physicists by (1) providing a consensus‐based framework for assessing the competence and independence of trainees in residency programs based on their demonstrated clinical capability, (2) facilitating feedback to residents based on their independence and competence in performing routine clinical tasks expected of a practicing medical physicist, and (3) providing a metric for programs to assess the success of their training programs and compare to other residency programs. This article describes EPAs and their potential use for assessment in medical physics residency programs.

## 1 INTRODUCTION

The goal of this article is to introduce medical physicists and medical physics educators to the concept of entrustable professional activities (EPAs). We aim to explore how EPAs can be used to standardize and enhance residency training and evaluation across programs, to understand their use as a strategic tool for resident evaluation and feedback, to review guidelines on how to create and describe EPAs for medical physics residencies, and to disseminate details regarding the development and assessment of EPAs to medical physicists. We hope that this work may increase interest in EPAs within our field and prompt future development, including the rigorous definition of EPAs for medical physics residencies.

## 2 WHAT ARE EPAS?

Competency‐based medical education (CBME) is a learner‐centered educational approach that emphasizes whether a learner has gained the skills required to become a capable professional upon graduation. It de‐emphasizes the focus on a prescribed training process and duration and aims for increased accountability and flexibility.^1^, ^2^, ^3^, ^4^ CBME centers on the learner's progress and development by identifying the synthesis of knowledge, skills, and attitudes that define a certain level of competency.^3^, ^5^ Hence, learner outcomes are used to guide curricular development in contrast to traditional methods that focus on knowledge objectives and defines sufficient training as clinical exposure to a particular number of cases and/or training for a certain period of time. The evolution towards CBME was motivated by the recognition that individuals may achieve competency at different speeds and via variable pathways and that time spent in training does not guarantee independent competency.^6^ Of note, CBME is not universally supported and its critics note that it could oversimplify professional competence, too quickly focusing on measurable outcomes instead of the development of higher‐order skills.^7^ It has nevertheless gained global traction due to a focus on learner and patient outcomes and accountability, and these criticisms have been considered as CBME has evolved.^8^

**FIGURE 1:**
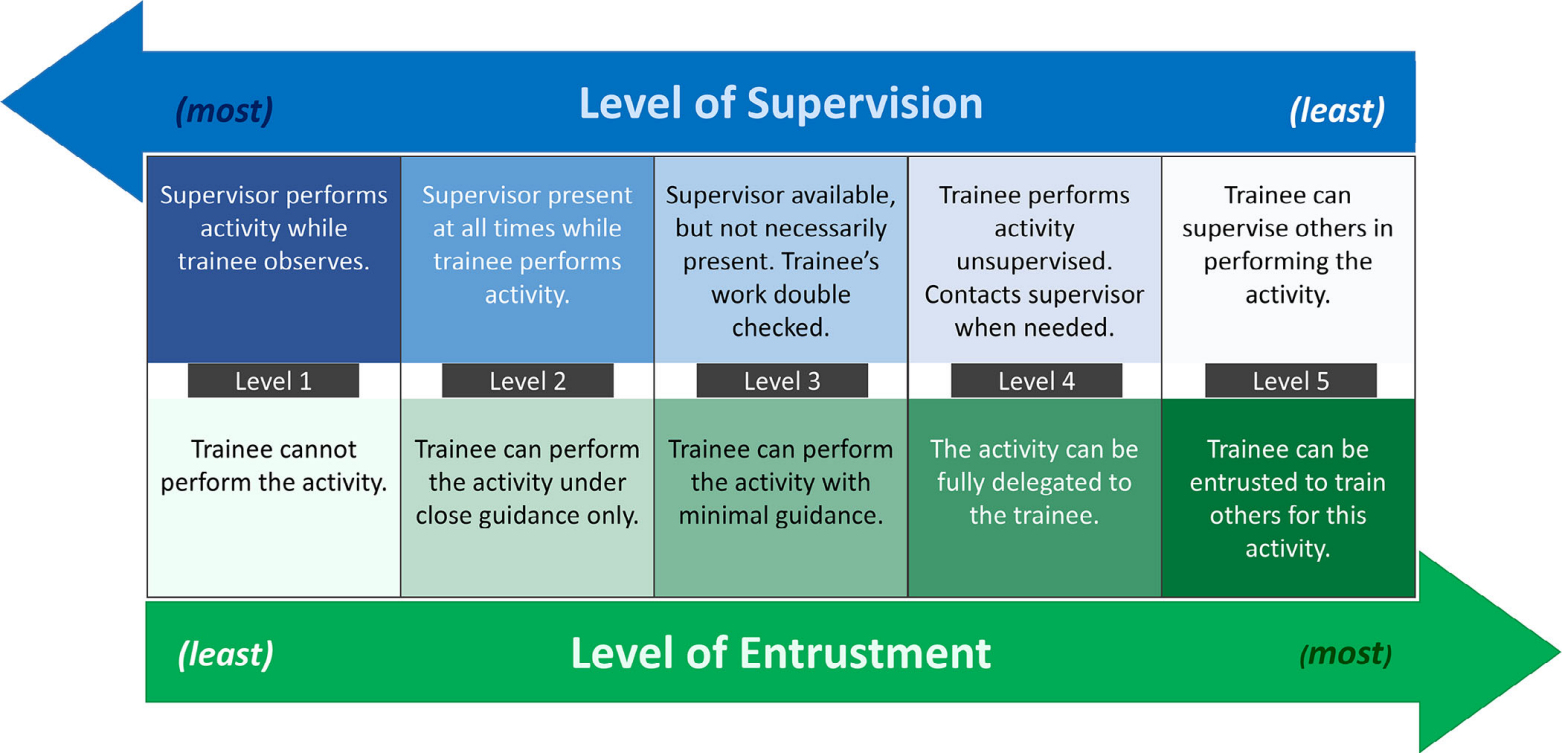
As the level of supervision required by the trainee decreases, the level of entrustment to perform the task independently, correctly, and safely increases. Each level shows progression through training, with level 4 being expected to achieve independent practice.^13^

EPAs were first introduced by Ten Cate in 2005, when a need to assess competencies was identified as a critical step to the successful implementation of CBME.^9^ EPAs are defined as “a unit of professional practice that can be fully entrusted to a trainee, once he or she has demonstrated the necessary competence to execute this activity unsupervised.”^10^ An EPA is a specific and focused clinical task that has a clear beginning and end and can be independently executed to achieve a defined clinical outcome. It requires the application of knowledge, skills, and/or attitudes acquired progressively through training and generally involves multiple domains of competence. EPAs were introduced to provide a transparent assessment framework for CBME grounded in day‐to‐day clinical activities that can be easily observed and measured.^6^, ^11^ Given that both competencies and EPAs are outcomes‐based, understanding the distinction between them is important. Competencies describe the abilities that a person should possess, while EPAs describe the essential healthcare work output (clinical task) that a learner should be able to perform independently. Therefore, successful completion of an EPA requires the integration and practice of several competencies relevant to the clinical task.^12^ EPAs further operationalize CBME by creating proficiency stages for the learner based on level of entrustment from the educator. The learner is gradually given increased responsibilities as they progress through the entrustment continuum.^13^ Since it is not guaranteed that the learner will be able to perform independently with each increment of responsibility, the autonomy is offered in steps to ensure patient safety. The entrustment level is inversely proportional to the supervision level needed for the trainee, which provides an intuitive and straightforward way to quantify entrustment (Figure 1). However, the evolution of trainee proficiency and progress along the entrustment continuum cannot be properly assessed unless the opportunities for observation from a supervisor are plentiful. For this reason, it is important that the activities selected as EPAs not only represent what makes a competent professional but also occur with enough frequency that trainees have the opportunity to perform them often under observation by a trainer.

Additional CBME frameworks, such as milestones, can be used in conjunction with EPAs to chart the developmental path of trainees in greater detail.^14^, ^15^, ^16^ Milestones describe the level of knowledge, skills and attitudes a learner is expected to achieve as they progress through their training.^17^ Milestones were implemented in US Graduate Medical Education (GME) by the Accreditation Council for Graduate Medical Education (ACGME) beginning in 2013,^18^ and they have been used independently of EPAs. Integrating these frameworks by mapping competencies and milestones to EPA entrustment decisions can clarify resident performance evaluations and allow for a more structured and specific feedback discussion on what the learner has accomplished and what needs further development.^14^ For example, milestones can be incorporated as part of an EPA; if an EPA is not fully achieved, identification of the unmet milestone can direct future learner efforts.

The implementation of EPAs allows trainees to be assessed through more regular low‐stakes evaluations (formative assessments—e.g., identifying and discussing a trainee's level of entrustment throughout the course of a rotation) that provide an opportunity for more immediate feedback as opposed to a single higher‐stakes evaluation per module (summative assessments—e.g., an end‐of‐rotation exam). As a result, these smaller, more frequent evaluations can become “assessments *for* learning” rather than “assessments *of* learning,” which may help learners further develop in their professional role by receiving individualized, timely feedback.^19^

## 3 WHERE ARE EPAS CURRENTLY USED, AND WHAT HAVE BEEN THE OUTCOMES?

EPAs have been successfully implemented within healthcare education, allied health professional education, medical school education, and post‐graduate medical training. As an example, in 2014, the Association of American Medical Colleges (AAMC) established 13 Core EPAs expected of all graduating medical students prior to transitioning into residency.^16^ The rationale for this approach and a description of these core EPAs are provided on the AAMC website, along with a curriculum developer's guide, a faculty and learners’ guide, and related toolkits and publications.^20^ Results of a pilot study to test the feasibility and impact of these EPAs demonstrated the ability to help faculty provide “useful, relevant, and specific formative feedback” to students.^21^, ^22^

EPAs have played a growing role in the training infrastructure of numerous medical specialties. For example, guidance on the development and use of EPAs is described on professional websites such as the Alliance for Academic Internal Medicine (in the United States),^23^ the Association of Family Medicine Residency Directors (in the United States),^24^ the American Board of Pediatrics,^25^ the American Board of Surgery,^26^ and the Royal College of Physicians and Surgeons of Canada.^27^

Large‐scale implementation of EPAs for medical education has occurred in both the Netherlands and Canada. These national efforts can serve as examples of how this new structure has been deployed in GME and its reported benefits and challenges thus far. In 2014, the Dutch Association of Medical Specialists (DMAS) launched the “Individualizing Postgraduate Medical Training” project, replacing fixed‐ length medical training with time‐variable individualized EPA‐based programs in all medical specialties.^28^ By 2019, 30 programs had implemented the new structure yielding an average training length reduction of 3 months.^28^ EPAs are currently also being developed for medical physics residency training in the Netherlands (personal communication, 2023).

The Canadian GME system offers a well‐documented example of the implementation of EPAs. The Royal College of Physicians and Surgeons of Canada, which plays a central role in oversight, accreditation, and certification in post‐graduate specialty training there, has created the Competence By Design initiative for medical education based on their implementation of the CanMEDS 2015 framework.^27^ This medical education plan represents a hybrid model in which CBME is implemented within a time‐based training structure. Competence By Design divides each residency into four stages of training (transition to discipline, foundations of discipline, core of discipline, transition to practice) with associated EPAs and milestones that a resident must fulfill before progressing to the next stage.^29^ Since initial implementation, residents and supervisors have overall reported an increase in the quality of feedback and better tracking of resident performance, but with increased workload and additional administrative burden from frequent observations.^30^, ^31^, ^32^, ^33^, ^34^ Although the increased frequency of observations and associated formative evaluations is meant to create a low‐stake assessment environment, supervisors or trainees may not always perceive this as such.^35^, ^36^, ^37^, ^38^ A need for further education on CBME principles and implementation, and better tools for assessment and activity tracking has also been identified.^39^, ^40^, ^41^

Current initiatives are underway to formally incorporate EPAs into the educational infrastructure of Radiation Oncology training pathways. The 4^th^ edition of the European Society for Radiotherapy and Oncology (ESTRO) core curriculum for radiation oncology/radiotherapy was published in 2019 and identifies 14 EPAs along with the competencies necessary to perform them.^42^ More recently, the Radiation Oncology Education Collaborative Study Group (ROECSG) developed a US radiation oncology curricular framework which identifies seven content domains and developed 52 EPAs within four competency‐based phases.^43^

## 4 CAN EPAS BE USEFUL FOR MEDICAL PHYSICS?

Currently, clinical medical physics residency training lacks a national assessment standard that would allow educators and future employers to compare the preparation and competency of individual medical physicists graduating from a residency training program. At this time, there is no CAMPEP‐required assessment standard for medical physics residency training. While board certification provides a standardized measure of competency for individual medical physicists, this evaluation is not available in time for a training program to address any shortcomings or for employers to assess new residency graduates at the time of hiring decisions. EPAs represent a more direct and timely feedback loop, provide public transparency, and promote accountability.^11^ Medical physics residency programs already commonly incorporate assessment mechanisms, such as rotation examinations. However, while some forms of examination may test knowledge, EPAs directly evaluate the activities we want our trainees to be able to perform independently and safely as clinical medical physicists. They would ideally be standardized to provide better uniformity across our national training infrastructure although an individual program could elect to use locally developed EPAs for assessment until a standardized consensus set is developed. Thus, implementing EPAs may substantially enhance our ability to produce higher quality and more uniformly competent trainees upon graduation.

Curriculum development for medical physics residency programs is guided by the CAMPEP standards and AAPM Report Number 249: *Essentials and guidelines for clinical medical physics residency training programs*, which provide requirements and guidelines, respectively, describing specific competencies that residents should acquire during a training program.^44^, ^45^ The development of EPAs would complement these guidelines by bringing together stakeholders to identify clinical activities that combine and operationalize these competencies into a consensus set of abilities that trainees should possess in order to be deemed prepared to practice medical physics independently upon graduation. A comprehensive set of EPAs for therapy, diagnostic, and nuclear medical physics would provide a broad and structured overview of training that mirrors the tasks performed by professionals in the field. Additionally, this would facilitate measurement of a trainee's ability to apply their knowledge to demonstrate an ability to perform specific clinical tasks. This would reduce variability in the proficiency level of graduating residents from different programs and give prospective employers a better sense of the minimum level of performance and independence to expect from a new graduate. Furthermore, this would create an environment where trainees could be safely allowed to practice with progressively less supervision during residency, which may better prepare them for independent medical physics practice upon graduation.^28^

Establishing a framework with which to evaluate our trainees across the field could improve the quality and consistency of our educational and training programs. In addition, aggregate EPA results could be used by programs as a metric to infer how well their training program prepares their graduates. These aggregated results might also be used to enable comparisons across different programs (e.g., trainer expectations and resident preparation).

## 5 PATH TO IMPLEMENTATION IN MEDICAL PHYSICS

To consider using the EPA model as an educational structure for medical physics residency and to determine an effective introduction into our field, it is useful to learn from the approaches and challenges described by others. First, there are many aspects that contribute to the success of EPA‐based training, but at baseline, if the EPAs selected for a specialty do not properly reflect the capabilities necessary for professional practice or are ill‐defined, their value as a reliable tool to evaluate professional competence is diminished.^46^, ^47^ Therefore, robust identification and definition of EPAs is crucial to the success of the project. Second, with the implementation of EPAs comes the need for an educational culture shift within each program and in the community of practice.^48^, ^49^ Whether EPAs are implemented exclusively using a CBME format enabling time‐independent training or using a hybrid model with competency‐based training embedded into a fixed‐time structure, their successful deployment hinges on buy‐in from and investment by stakeholders and thorough education on EPA principles for all participants, including trainees.^39^, ^50^, ^51^ A move away from time‐dependent training length is not necessary to the successful implementation of EPAs, would require a change in CAMPEP accreditation standards, and may not be feasible in the clinical setting. It takes time, training, and support for programs and individuals to transition to the new paradigm, which leads to the third point: widespread adoption of EPA‐based education requires a commitment from institutions for both time and resources. This includes time and resources for training, tool development, curriculum design and implementation, faculty development, and a long‐term commitment to continuously iterate, evaluate, and update the process to ensure success and relevance with changing practices.^28^, ^51^, ^52^, ^53^ For this change to be realized, educators will need to be supported and prepared to lead, to share resources, and to advocate for and facilitate structural change.^48^, ^49^

### 5.1 How to select EPAs

Most descriptions of EPAs in the literature present a similar high‐level pathway for EPA selection^46^: (1) gather a group of experts, (2) establish a list of EPAs and respective descriptions, (3) use some method of iterative consensus building to ensure EPAs and definitions are appropriate/adequate, and (4) vet EPAs and descriptions by seeking input/feedback from members of the professional group. Gathering a diverse group of experts increases the likelihood that the resulting list of EPAs is sufficiently comprehensive and that the selected activities are well suited for the EPA format. These experts should have intimate knowledge of the breadth of what is clinically observable for a given profession and a variety of backgrounds (community practice/academic medical center, various degrees of specialization, physicians and education experts alongside medical physicists).^46^ Commonly, a Delphi approach is used for consensus building. This method involves identifying an initial list of EPAs (often by a smaller expert group), providing an opportunity for individual and/or anonymous feedback from a larger group, making changes to the EPA list in response, and iterating until consensus is reached.^43^, ^46^

Guideline documents, such as AAPM medical physics practice guideline (MPPG) 10.a: *Scope of practice for clinical medical physics* could be used as a starting point for identifying and defining EPAs, while the CAMPEP standards and AAPM Report 249 may be helpful resources to inform the scope of EPA descriptions.^44^, ^45^, ^54^ Following the process described in the literature, EPAs in medical physics could be initially developed by a small group in each specialty (e.g., therapy, diagnostic, nuclear medicine), possibly in consultation with education experts, and later vetted by relevant professional societies prior to being piloted.^28^ The development of medical physics EPAs is beyond the scope of this article because such development is complex and requires iterative refinement and consensus among a broad group of stakeholders. However, it is noted that in medical physics, technology develops rapidly, and some residency programs can offer training in new modalities or techniques that others cannot. EPAs should be so fundamental that any CAMPEP‐accredited program should be able to offer the training, and they should be aligned with CAMPEP standards. EPAs could potentially be structured to expand to other modalities if available, and some EPAs could be deemed optional. In some cases, EPAs can be written without regard to specific technology, and can evolve with the field. For example, EPAs developed for breast radiology have several modality‐neutral EPAs with sub‐parts for different imaging modalities.^55^, ^56^

### 5.2 How to describe EPAs

Ten Cate recommends that EPA descriptions contain the eight sections listed in Table 1.^5^ Note that sections 1, 2, 3, 4, 5 and 8 (title; specification and limitations; potential risks related to failure modes; relevant competency domains; knowledge, skills, attitudes, and experiences required to elicit entrustment; frequency of practice to maintain competence) can be standardized across programs, but Sections 6 and 7 (sources of information for assessing progress and summative entrustment; expected entrustment/supervision level at learner's stage) may benefit from program‐specific descriptions to account for different clinical environments and local rules/regulations. Each EPA should be standalone and have minimal overlap with others. An advantage of constructing EPAs is to provide a shared framework among the various stakeholders to inform competency‐based education and provide standards for the scope of practice.^5^ In addition, an effective EPA description would guide both trainers and trainees on how to achieve entrustment by identifying learning goals, activities, and observation opportunities for assessment, and provide a clear structure on which to provide constructive feedback.^5^, ^13^ An EPA description may additionally include specification of multiple assessors or multiple assessment instances before it can be considered complete, depending on the complexity, essentiality, and clinical frequency of the task. Robust EPA definitions and descriptions can inform teaching content and guide the clinical learning opportunities that the training program should provide. They further suggest to trainees which learning opportunities they need to seek (e.g., additional commissioning opportunities or multiple annual linac QA practice) in order to achieve competence for a given activity.^13^

**TABLE 1:** Recommended sections to include when describing EPAs (adapted from Ten Cate O, Taylor DR. The recommended description of an entrustable professional activity^5^).

Section	Description
1. Title	Clear and indicative of the activity. Unambiguous to all stakeholders. It should be similar to an item in a job description (i.e., “Evaluating the quality of a treatment plan” or “Managing an imaging quality assurance program”). It should not be worded as an educational objective.
2. Specification and limitations	Clear description of the elements and scope of the activity, including the context under which the resident is expected to be able to perform (i.e., “Excludes patients with prior radiotherapy treatments” or “Understands how quality assurance programs can and should differ by imaging modality”).
3. Potential risks related to failure modes	Description of the consequences to patient care if the activity is not performed properly, to help supervisors with difficult entrustment decisions.
4. Relevant competency domains	List the competency domains (i.e., content expertise, communication skills, professional behavior, etc.) the learner will need in order to perform the activity. This serves to connect the EPA to existing competency frameworks, when available.
5. Knowledge, skills, attitudes, and experiences required to elicit entrustment	Identify the knowledge, skills, attitudes, and experience the learner must have to warrant decreased supervision. This section also gives learners a sense of the expectations and criteria for advancing through the entrustment continuum.
6. Sources of information for assessing progress and summative entrustment	Describe the information to be used for assessment. This should include more than one source (i.e., observe resident doing plan checks, present different scenarios to resident and have them describe what they would do and why, have residents perform practice chart checks and review the results, etc.). Minimum number of assessments needed to reach a reliable summative entrustment decision should also be included. (A summative entrustment decision is a formal decision about how much supervision a learner needs.) Consider the breadth of context, the number of assessments needed to confidently determine whether the resident is consistently performing at the expected level, the stakes of entrustment, etc.
7. Expected entrustment/supervision level at learner's stage	Using a scale like that presented in Figure 1, indicate the level of supervision/entrustment expected as the learner progresses through training.
8. Frequency of practice required to maintain competence	Indicate if the entrustment decision will expire after a certain time period if the activity has not been performed.

Appendix S1: *Examples of EPAs in Radiation‐Related Fields* includes two examples of EPAs currently being used in radiation‐related disciplines. The first example is of an EPA developed for Breast Radiology.^55^, ^57^ The second is an EPA developed for Radiation Oncology under the Competence By Design initiative.^27^, ^58^ In these and other EPA examples, though the actual task (typically the EPA title) should be specific and focused, the EPA descriptions may be more lengthy to fully describe component competencies, references, and activities, as an aid in trainee assessment and curriculum design.^25^, ^55^, ^56^, ^59^, ^60^ Although the EPA description format detailed in Table 1 is recommended by education experts, EPA descriptions in different fields and subspecialties may vary from this format.

### 5.3 How to evaluate our trainees based on EPAs

EPAs provide a framework for evaluating trainees. Consciously or unconsciously, clinical educators ask themselves, “Do I trust this learner to do that clinical task?” By defining a specific EPA, this intuitive evaluation process becomes explicit and concrete; consequently, the trainer may also be alerted to any possible biases in the process. There are multiple factors that contribute to gauging entrustability. A formulation compiled from publications in medical education literature suggests five traits that are the ″ingredients of a rich entrustment decision.^61^ They are shown in Table 2. Evaluating entrustment involves more than whether the trainee knows the underlying medical physics facts and the specific steps to complete the clinical task. It includes whether the trainee can be trusted to honestly present the results, own up to any mistakes, complete the work in the required timeframe, and know when to seek help or how to react when something goes wrong. In this way, it incorporates feedback on trainee attitudes (along with knowledge and skills) in a way that has proven challenging in our current approaches. Attitudes relevant to entrustment include integrity, honesty, patient‐focus, and team player. Additional factors influence the educator's level of trust, including the task (complex vs. simple), the workflow (fast‐paced vs. less intense), and the personal attributes of the supervisor in regards to where they lie along the spectrum from skepticism to trust.^62^ Beyond competence, EPAs and entrustability also consider the consequences of the trainee's work output for the particular clinic and patient.

**TABLE 2:** Components of enriched entrustment decisions.^61^

Component	Definition
Capability	Specific knowledge, skills, experience, situational awareness
Integrity	Truthful, benevolent, patient‐centered
Reliability	Conscientious, predictable, accountable, responsible
Humility	Recognizes limits, asks for help, receptive to feedback
Agency	Proactive toward work, team, safety, personal development

The key to the successful application of EPAs is to associate the assessment of the learner with the decision that they can be entrusted with responsibility for clinical tasks.^13^ Educators should explicitly discuss the concept of trust with their learners, who tend to focus on competencies or grades instead of trust. By directly referencing the EPA, the educator sets expectations for trainees and explains how all five components contribute to trust decisions. Additionally, the program structure should identify periodic assessment and performance‐based feedback time points and use the EPAs as the framework for giving credible, effective feedback to the trainee in the context of the clinical activity. Case‐based discussions can be used to talk through rare scenarios (e.g., what would you do if the output is off by 5% on a monthly linac QA?, or what would you do if the air kerma rate on an interventional unit is > 10 R/min at 30 cm from the detector input?) in order to build trust in the trainee's performance in a future situation.

Educators should be able to describe a typical entrustment continuum and identify when a learner has reached a plateau to aid them in navigating past it. If the learner has not yet demonstrated entrustability, the educator should reflect on whether additional observation of the learner in action would aid in cementing the decision or whether additional training/practice opportunities under supervision are needed. The learner should also be provided with opportunities for self‐reflection regarding their entrustability. These opportunities for reflection are incorporated through frequent formative assessments regarding the learner's place on the entrustment continuum.

The concept of levels of supervision, used to anchor entrustment decisions in EPAs, appears in AAPM MPPG 3.b: *Levels of supervision for medical physicists in clinical training* in a similar form, though completely independent of EPAs.^63^ This document defines the levels of supervision for medical physicists in clinical training and provides a language and understanding of minimal standards of supervision for trainees and new medical physicists.^10^ Competency in AAPM MPPG 3.b is defined as “the demonstrated ability to independently perform the medical physics‐related task or function.” Supervision is defined as the “oversight of and acceptance of responsibility by the QMP [Qualified Medical Physicist] for the medical physics‐related work performed by a Trainee or Medical Physics Student.” AAPM MPPG 3.b introduces three supervision levels.^63^ Many EPA frameworks utilize a five‐point scale^10^ with similar language describing the level of supervision needed based on the level of trust. The five‐point entrustment scale is shown in Table 3, along with the MPPG 3.b supervision scale. In EPA entrustment scales, the three levels of AAPM MPPG 3.b are supplemented by a pre‐level (observe only) and post‐level (trusted to train others).

**TABLE 3:** Mapping of entrustment and supervision scales to MPPG 3.b supervision scales.^13,63^

EPA entrustment and supervision scale	AAPM MPPG 3.b supervision scale
Not allowed to practice EPA: Inadequate knowledge or skill; not allowed to observe Adequate knowledge, some skill; allowed to observe	
2. Allowed to practice EPA only under proactive, full supervision: As coactivity with supervisor With supervisor in room read to step in	Personal supervision: A QMP must exercise General Supervision (described below) and be present in the room during the performance of the procedure.
3. Allowed to practice EPA only under reactive/on‐demand supervision, with supervisor: immediately available, all findings and decisions double‐checked immediately available, key findings and decisions double‐checked distantly available (e.g., by phone), findings and decisions promptly reviewed	Direct supervision: A QMP must exercise General Supervision (described below) and be present in the facility and immediately available to furnish assistance and direction throughout the performance of the procedure. It does not mean that the QMP must be present in the room when the procedure is being performed.
4. Allowed to practice EPA unsupervised: With remote monitoring (e.g., next day check‐in for learner questions) Without monitoring	General supervision: The procedure is performed under a QMP's overall direction and control but the QMP's presence is not required during the performance of the procedure. Under General Supervision, the training of the personnel who actually performs the procedure and the maintenance of the necessary equipment and supplies are the continuing responsibility of the QMP.
5. Allowed to supervise others in practice of EPA.	

Assessing competency via trust relies on the quality of the relationship between the learner and educator.^6^ A thoughtful approach to building trust and managing conflict will be needed.^64^, ^65^ The relationship between educator and learner may be strengthened using consistent pairings for longer periods of time. If this is not possible, using longitudinal portfolios, where supervisors can record the learner's performance and their experience with the learner during their interaction, can help convey information across the training period.^13^ The intuitiveness of EPAs comes from the fact that educators are encouraged to listen to and understand their gut feelings, which may result from unconscious processing of past experience with the trainee. When the educator reflects on why they may not yet trust the learner, they should consider information regarding the learner's integrity, reliability, and humility in addition to their competence. Multiple observers are advantageous to overcome potential limitations of the assessment skills and bias of the educator. Clinical Competency Committees are utilized within GME programs for similar reasons.^27^, ^66^ To avoid issues with inconsistencies in evaluations between supervisors, it is important to clearly define the expectations for each trainee level. This is where the introduction of milestones along with EPAs may be useful.^14^, ^40^ It is also essential to consistently document the progress of each learner in the program. Aggregate data will provide useful metrics for program evaluation, identifying weaknesses in the program's training structure and informing the logical sequence of learning opportunities.

## 6 EXPECTED CHALLENGES

Though implementation of pure outcomes‐based CBME is inconsistent with fixed‐duration residency training, we can still effectively apply CBME concepts, such as EPAs, within our current model to improve the quality of our training. Initiatives like the Royal College of Physicians and Surgeons of Canada's “Competence by Design” provide a model for combining time‐based and outcomes‐based learning.^27^ Effective implementation of CBME presents a shift from the traditional formulation, structure, and evaluation methods of training programs. It raises the questions of “What does it mean to be competent?” and “How do you assess competence?” as part of the curriculum design. These and other challenges of implementing CBME are summarized here to highlight their potential impact on medical physics training.^53^, ^67^

### 6.1 Educational and assessment culture change

Culture change takes time, and such a shift in educational practice would require adaptability and a system that enables iterative evaluation and modification of the performance of the new approach. Both faculty and trainees would benefit from support and training to thrive in the new paradigm. A continuous faculty development plan that includes training of faculty and helps them adapt their skill set to the new evaluation framework would promote community building to facilitate culture change.^52^ Trainees would need a good understanding of the CBME model, the purpose behind the new assessment scheme, and the value of longitudinal assessment and high‐quality feedback for their training.^51^ If these aspects are not clearly conveyed, trainees may misconstrue every low‐stakes assessment as a high‐stakes assessment instead of an opportunity to receive constructive feedback and grow in their clinical practice.^38^ At the same time, it will be important that faculty embody the principles behind CBME and learn how to provide specific, actionable feedback to reinforce the utility of formative evaluations whenever they observe residents performing an EPA.

### 6.2 Defining EPAs in medical physics

Distilling a profession into discrete clinical activities that embody what it means to be a competent professional is challenging. Such clinical activities must be defined in a clear, descriptive, accurate, and measurable way, and should be widely accepted by the community. Hasty implementation of suboptimal EPAs diminishes their utility as performance assessment tools.^46^ Achieving consensus will require an investment of time and resources and an iterative development process. However, undertaking such a project would provide clarity regarding training goals and a roadmap to consistent and meaningful evaluation in residency programs. This could help achieve consistent performance standards across residency programs and provide the field with a more explicit set of expectations for graduating residents. Once consensus EPAs have been established, they could potentially facilitate new staff training or credentialing efforts.

Due to technological advances, medical physics is an ever‐changing field. EPAs would have to be revised and updated regularly as practice evolves. However, to diminish the frequency in which these updates are needed, it will be important to define EPAs so their focus is on underlying principles and to understand the impact on patient care quality, rather than the specifics of how the activities are conducted with a given piece of equipment. Thus, it is important to remember that the resident would need additional support and an adjustment period before they are entrusted to independently perform an EPA when working with unfamiliar equipment or new workflows at a new facility.

### 6.3 Evaluating residents with EPAs

As previously discussed, one of the advantages of EPAs is the intuitive evaluation scale based on how much the supervisor trusts the resident to perform the given activity safely and independently. While easy to adopt by supervisors, making reliable judgments of entrustability requires significant consideration. After all, as pointed out by Gingerich, ″trust can be assumed, inferred, felt, created, discovered, earned and lost, but not observed.^68^ We have already discussed that additional factors influence the educator's level of trust, including the task (complex vs. simple), the workflow (fast‐paced vs. less intense), and the personal attributes of the supervisor in regard to where they lie along the spectrum from skepticism to trust.^69^ These judgments are almost certainly also subject to the same implicit biases seen in resident interview evaluations and elsewhere.^70^

Tools that decrease bias and increase reliability would need to be developed. Establishing a valid evaluation scale for EPAs should help resolve these issues as it will make an assessor's evaluations more consistent.^68^ Even with such tools in place, assessors should continuously reflect on whether factors unrelated to the observed resident performance are affecting their evaluation. These can include elements such as recent personal events, stress from high workload, prior interactions (with that resident or others) that may impact their judgment, etc. In cases where the assessor does not think they can provide a fair evaluation, the evaluation should be rescheduled or an alternative person should step in to perform the evaluation when possible. By making entrustability explicit, it would be easier to identify potential biases such as those listed above.

Ultimately, the evaluation of “do you trust this resident to perform the task safely and independently?” is a very clinically meaningful measure as long as the entrustment decision has been made fairly and takes into account the impact of that task on patient care. Although perhaps not officially, this type of evaluation is something we already do routinely. Adopting the EPA framework would provide a more systematic, consistent, and methodical way to formalize and better apply this meaningful metric in our training.

### 6.4 Administrative burden

Implementation of EPAs will pose an additional administrative burden and resource investment. The Canadian Competence By Design program, a highlighted model for EPA implementation, benefits from funded administrative support, training, tools, and implementation on a broad scale. The setting of medical physics residency training—non‐physician training which is already challenging to fund and sometimes outside a medical school setting—may be a more challenging environment in which to implement EPAs. Proper integration of EPAs into a residency curriculum will require each program to critically evaluate their training structure to determine if the given EPAs align with their current curriculum design, and if not, identify and execute the necessary changes for implementation. It will also involve training to ensure educators are familiar with the EPA structure and evaluation style. Assessing level of entrustment requires multiple observations and, ideally, multiple observers to minimize the effects personal bias might introduce into determining entrustment. Given that there may be an increase in the number of evaluations and data acquisitions, this would also pose an additional burden on the individual programs that needs to be considered and proactively addressed to minimize impact.^69^

Harnessing inter‐institutional collaborations could alleviate some of these burdens. For example, EPA development, implementation, and testing could be carried out by a national cooperative group of educators. Education of trainers on the future use of EPAs could also be accomplished through educational workshops sponsored by professional societies. Mentorship could be provided to faculty as they incorporate EPAs into their residency training programs.

## 7 CONCLUSION

EPAs are a useful tool that operationalizes CBME principles. They shift the focus from a prescribed training process and/or number of cases as a surrogate for competence to assessing residents’ entrustability for clinical tasks that embody the profession.

Based on our literature review, the following points summarize the potential value of incorporating EPAs in our training programs and factors to consider for their implementation:

- EPAs could help cement competency‐based training in medical physics residency programs.
- Achieving a consensus set of EPAs to describe the medical physics profession for each specialty could establish a national framework to better standardize training and evaluation of residents.
- The group of activities selected as EPAs should describe what it means to be a competent clinical medical physicist and clearly depict the capabilities necessary for professional practice. Not every task a medical physicist performs needs to be included as an EPA.
- Activities selected as EPAs should be specific, focused, well‐defined, and readily observable so there can be enough observations to allow for formative assessment and constructive feedback prior to summative evaluation.
- Effective EPA descriptions should trace a path to entrustment by identifying learning goals, activities, and observation opportunities for assessment, and setting a structure for providing constructive feedback.
- EPAs leverage the intuitive evaluation of whether a trainer trusts the trainee to perform a given clinical task. To minimize bias in these evaluations, there should ideally be many observation points with as many different trainers as is feasible, and trainers should be well‐versed in the potential sources of bias for these types of evaluations.
- Before, during, and after implementation of EPAs, both trainers and trainees will need on‐going education in CBME concepts, the EPAs to be used during the residency program, and how to use them.
- Development of EPAs should occur within a multi‐institutional collaboration that involves all stake‐holders, including physicists, trainees, other team members (e.g., physicians), and education experts.

Although adoption of EPAs would require a large investment of time and resources, their implementation would help standardize medical physics residency training across programs, promote better feedback and clearer assessments for both faculty and trainees, and ensure a more consistent performance level amongst graduating residents. They would help create a consensus standardized set of clinical activities that residents should be capable of performing safely and independently by graduation.

## AUTHOR CONTRIBUTIONS

All authors listed contributed to the concept, discussions, and drafting of the study to present the educational recommendations and conclusions. Authors contributed to and approved the final version of the manuscript.

## CONFLICT OF INTEREST STATEMENT

The Chair of the Working Group on Entrustable Professional Activities for Medical Physics Residents has reviewed the required Conflict of Interest statement on file for each member of the Working Group on Entrustable Professional Activities for Medical Physics Residents and determined that disclosure of potential Conflicts of Interest is an adequate management plan. The members of Working Group on Entrustable Professional Activities for Medical Physics Residents listed below attest that they have no potential Conflicts of Interest related to the subject matter or materials presented in this document: Laura Padilla, PhD, Christina L. Brunnquell, PhD, Jay W. Burmeister, PhD, Derek W. Brown, PhD, Kristi R. G. Hendrickson, PhD, Hania A. Al‐Hallaq, PhD.

## Supporting information



Supporting Information
