# Supplementary material for: AAPM WGEPA Report 437: An introduction to entrustable professional activities for medical physics residency education
Source: J Appl Clin Med Phys. 2025 Oct 15;26(10):e70198. doi: 10.1002/acm2.70198 (PMC12527643; doi:10.1002/acm2.70198)
Supplement: Supplementary file 1 — Supporting Information [file ACM2-26-e70198-s001.docx]

**Appendix 1/Supplementary File 1: Examples of EPAs in radiation-related fields**

The following examples illustrate EPAs used in the fields of Radiology and Radiation Oncology. These specific examples were selected to illustrate how different programs have adopted different formats for their EPA descriptions.

A1.A. Radiology

The following provides an example of one of the EPAs developed for Breast Radiology.^49–51^ The materials developed for this EPA include the EPA description, an “Assessment Guide” to facilitate evaluation of the EPA, and a “Teaching Tips” sheet to guide teaching, all of which are included in this supplementary material.

The material reproduced below is from the 2019 *Breast Radiology Entrustable Activity Supervision Tool*^49-51^. Authors: Monica Sheth, MD; Ryan Woods, MD; Katherine Klein, MD; Prisciplla Slanetz, MD; Alice Fornari, EdD; Petra Lewis, MBBS.

When compared to the description sections listed in Table 1, this EPA description does not explicitly include “Potential risks related to failure modes,” and additionally includes a section with all suggested references for the trainee to review and consult related to this EPA. The rest of the sections can be directly mapped to those listed in Table 1.

**EPA1a-BR Worksheet**

| **Title** | Identifying and managing abnormalities on screening examinations  - EPA1a: Mammography |
| --- | --- |
| **Description of Activity** | A radiologist involved in breast imaging must be able to identify abnormalities on screening examinations while adhering to Mammography Quality Standards Act and Program (MQSA) and determine the next steps in patient management.  The key function which define this EPA in regards breast screening examinations include:  ❏ Lists indications for each screening modality^1,12,13^  ❏ Understand technique, patient positioning, standard imaging views and study protocol^1,17,19^ ^20,21^  ❏ Differentiate technically adequate and inadequate studies^1^  ❏ Differentiate benign findings from those that warrant additional work- up6,9,21  ❏ Identify imaging artifacts and explain methods for correction^4,13^  ❏ Identify the normal and abnormal appearance of the breast after surgical procedures (reduction, augmentation, implants, breast conserving therapy, or mastectomy)^B,D,E,16^  ❏ Demonstrate the correct use of the BI-RADS lexicon terminology pertinent to the examination including assessment/management categories^A,1,6,9^  ❏ Report and communicate results with the patient, referring physician (including primary physician, oncologist, surgeon), and staff when indicated^F,11^  The key functions in regards to screening mammography include:   - Explain ACR/SBI screening mammography guidelines and how they vary from USPSTF guidelines (starting age, interval, etc, why different recommendations, general statistics)^2,3,12^ - Recognize the 4 breast density parenchymal patterns^C,7,8,18,22^ - Describe essential components of the mammogram report - Identify findings that warrant additional work-up (masses, calcifications, architectural distortion, asymmetries, focal asymmetries, global asymmetry, developing asymmetry, and abnormal lymph nodes)6,9,10,21,23 - Explain additional imaging needed in the diagnostic setting^3,5,21^ - Identify the normal and abnormal appearance of the breast after surgical procedures (augmentation, reduction, lift, implants; breast conserving therapy)^D,E,16^ - Identify artifacts on mammography and determine how to correct^4,13^ - *Counsel patients and referring physicians about supplemental screening modalities (US, MRI)^F,G,12^ - Understand the basic requirements of the MQSA as it pertains to screening mammography^14,15^ - *Calculate basic screening mammography audit metrics including recall rate, positive predictive value 1 (PPV1), and cancer detection rate^14,15^ |

|  | - Understand QA/QC requirements of analog and digital mammography^4^ Superscript indicate resources below which address the key function **Context:** Outpatient imaging center   **Targeted transition point:**  Depending on the institution - First month for screening mammography, second month for ultrasound, third month for MRI. Items marked * may be more suitable for month 3 of mini-fellowship or fellowship for some programs |
| --- | --- |
| **Mapping to Domains of Competence** | X Patient Care  X Medical Knowledge  X Systems-Based Practice  X Practice-Based Learning and Improvement X Professionalism  X Interpersonal and Communication Skills |
| **Competencies within each domain critical to entrustment decisions** | PC1: Reporting  PC2: Clinical Consultation PC3: Image Interpretation MK1: Diagnostic Knowledge MK2: Physics  MK4: Imaging Technology and Image Acquisition SBP6: Radiation Safety  SBP8: Informatics  P2: Accountability/Conscientiousness P3: Self-Awareness and Help Seeking  ICS1: Patient- and Family-Centered Communication ICS2: Interprofessional and Team Communication |
| **Suggested Resources**  (A) Article  (B) Book Chapter  (D) Document  (S) Slides  (W) Widget - interactive powerpoint  (V) Video | 1. [A Pictorial Review of Changes in BI-RADS 5th Edition](https://pubs.rsna.org/doi/10.1148/rg.2016150178) (A) [Slides](https://drive.google.com/open?id=1PJNvz0IwnvGbk_DpQJCbWEQIo2eVNVNo) 2. [Update on Imaging of the Postsurgical Breast](https://pubs.rsna.org/doi/10.1148/rg.343135059) (A) 3. [Hormonal Effects on Breast Density, Fibroglandular Tissue, and](https://pubs.rsna.org/doi/10.1148/rg.2018180035) [Background Parenchymal Enhancement](https://pubs.rsna.org/doi/10.1148/rg.2018180035) (A) 4. [Imaging of Breast Implant-associated Complications and Pathologic](https://pubs.rsna.org/doi/10.1148/rg.2017170025) [Conditions: Breast Imaging](https://pubs.rsna.org/doi/10.1148/rg.2017170025) (A) 5. [Breast Reconstruction: Review of Surgical Methods and Spectrum of](https://pubs.rsna.org/doi/10.1148/rg.332125108) [Imaging Findings](https://pubs.rsna.org/doi/10.1148/rg.332125108) (A) 6. [Maximizing Value Through Innovations in Radiologist-Driven](https://www.ajronline.org/doi/abs/10.2214/AJR.17.18410) [Communications in Breast Imaging](https://www.ajronline.org/doi/abs/10.2214/AJR.17.18410) (A) 7. [Training and Standards for Performance, Interpretation, and Structured](https://www.ajronline.org/doi/pdf/10.2214/AJR.14.13794) [Reporting for Supplemental Breast Cancer Screening](https://www.ajronline.org/doi/pdf/10.2214/AJR.14.13794) (A)   Mammography   - 1. [Screening](https://media.med.unc.edu/tec/coils/radiology/ViewsYouCanUseScreeningMammography/story_html5.html) [Mammography](https://media.med.unc.edu/tec/coils/radiology/ViewsYouCanUseScreeningMammography/story_html5.html) [- Presentation](https://media.med.unc.edu/tec/coils/radiology/ViewsYouCanUseScreeningMammography/story_html5.html) (V)   2. [Screening and Diagnostic DBT SBI White Paper](https://www.sbi-online.org/RESOURCES/WhitePapers/TabId/595/ArtMID/1617/ArticleID/595/Digital-Breast-Tomosynthesis-for-Screening-and-Diagnostic-Imaging.aspx) (A)   3. [ACR Practice Guideline for Screening and Diagnostic Mammography](https://www.acr.org/-/media/ACR/Files/Practice-Parameters/Screen-Diag-Mammo.pdf) (A)   4. [Optimizing Digital Mammographic Image Quality for Full-Field Digital](https://pubs.rsna.org/doi/10.1148/rg.2015150036) [Detectors: Artifacts Encountered during the QC Process](https://pubs.rsna.org/doi/10.1148/rg.2015150036) (A)   5. [Digital Breast Tomosynthesis in the Diagnostic Setting: Indications and](https://pubs.rsna.org/doi/10.1148/rg.2015140204) [Clinical Applications](https://pubs.rsna.org/doi/10.1148/rg.2015140204) (A) |

|  | 1. [Developing Asymmetries at Mammography: A Multimodality Approach](https://pubs.rsna.org/doi/10.1148/rg.2016150123) [to Assessment and Management](https://pubs.rsna.org/doi/10.1148/rg.2016150123) (A) 2. [Mammographic Breast Density: Impact on Breast Cancer Risk and](https://pubs.rsna.org/doi/10.1148/rg.352140106) [Implications for Screening](https://pubs.rsna.org/doi/10.1148/rg.352140106) (A) 3. [Breast Density: Clinical Implications and Assessment Methods](https://pubs.rsna.org/doi/10.1148/rg.352140134) (A) 4. [Interpreting One-View Mammographic Findings: Minimizing Callbacks](https://pubs.rsna.org/doi/full/10.1148/rg.344130066) [While Maximizing Cancer Detection](https://pubs.rsna.org/doi/full/10.1148/rg.344130066) (A) 5. [Mammographic Signs of Systemic Disease](https://pubs.rsna.org/doi/10.1148/rg.314105205) (A) 6. [Communication in Breast Imaging: Lessons Learned at Diagnostic](https://www.jacr.org/article/S1546-1440(16)31102-4/fulltext) [Evaluation](https://www.jacr.org/article/S1546-1440(16)31102-4/fulltext) (A) 7. [ACR Appropriateness Criteria Breast Cancer Screening](https://www.jacr.org/article/S1546-1440(16)30930-9/fulltext) (A) 8. [Digital breast tomosynthesis: Image acquisition principles and artifacts](https://www.ncbi.nlm.nih.gov/pubmed/30236642) (A) 9. [National Performance Benchmarks for Modern Screening Digital](https://pubs.rsna.org/doi/10.1148/radiol.2016161174) [Mammography: Update from the Breast Cancer Surveillance](https://pubs.rsna.org/doi/10.1148/radiol.2016161174) [Consortium](https://pubs.rsna.org/doi/10.1148/radiol.2016161174) (A) 10. [Audits, Benchmarks and Performance: What You Need to Know](https://www.sbi-online.org/Portals/0/Breast%20Imaging%20Symposium%202016/Final%20Presentations/4-10%20930am%20Burnside%20-%20Audits%20Benchmarks%20and%20Performance.pdf) (S) 11. [Implants on Breast Mammogram Widget](https://www.bookwidgets.com/play/TDTZ9E) (W) 12. [Breast Anatomy Quiz](https://www.bookwidgets.com/play/GDR9ZT) (W) 13. [Breast Density Quiz](https://www.bookwidgets.com/play/ADR97T) (W) 14. [CC Breast Anatomy Interactive tool](https://www.bookwidgets.com/play/RDR93G) (W) 15. [MLO Breast Anatomy Interactive tool](https://www.bookwidgets.com/play/4DR953) (W) 16. [Screening Mammography Need to Know Quick Review Document](https://drive.google.com/file/d/0BwGRo9UPfqPMWDB0RzNON0E4NnR0UEZ5VFJuRVlnQVcxZUlZ/view?usp=sharing) (D) 17. [Breast Density ACR Brochure](https://www.acr.org/-/media/ACR/Files/Breast-Imaging-Resources/Breast-Density-bro_ACR_SBI.pdf) (D) 18. [Introduction to Mammography](https://www.youtube.com/watch?v=dEdR4iOdLh0&feature=youtu.be) (V) |
| --- | --- |
| **Required knowledge, skills, attitude and behavior, and experience** | Knowledge   - Knowledge of imaging abnormalities on mammography - Knowledge of correct BI-RADS terminology to describe imaging findings. - Knowledge of markers of image quality. Skills - Skill in identifying abnormalities on mammography screening exams. - Skill in discussing results of imaging exams with patients, referring physicians, and staff   Attitude and Behavior   - Professional communication of screening exam results with patients, referring physicians, and staff.   Experience   - Screening mammography: 250-400 screening mammograms |
| **Assessment Information sources to assess progress and ground summative entrustment decision** | Knowledge Assessment:  RadExam Breast EPA1: Screening (under construction)  RadExam Breast EPA1: MQSA & Audit (under construction)  Review of interpretation of screening mammography with gradual decline in recall rate over time, if available (for example: 1st month: <50%; 2nd month: 30-50%; 3rd month: <30%)  5-10 informal case-based discussions per modality with attending radiologist |
| **Entrustment level of supervision to be reached at** | *Imaging studies should always be overread by an attending physician Residents: Indirect supervision (level 3) prior to graduation - ability to identify at least 50% of the abnormalities identified by the attending radiologist |

| **which stage of training** | Mini-fellows: Distant supervision (level 4) prior to graduation - ability to identify 50-75% of the abnormalities identified by the attending radiologist  Fellows: Trust to perform unsupervised (level 5) or to supervise others (level 6) prior to graduation (ability to identify 75-100% of abnormalities identified by the attending radiologist and ability to teach concepts to residents) |
| --- | --- |
| **Expiration** | 1 year after graduation |

*Modified from the work of Olle ten Cate, PhD

**EPA1a Attending Supervision Tool:** Screening Mammography

| Date |  |
| --- | --- |
| Trainee Name |  |
| R level | **▢**R1 **▢**R2 **▢**R3 **▢**R4 **▢**Mini-fellow **▢**Fellow |
| # Studies read |  |

EPA Supervision scale: Trainee is trusted to

1. Observe only
2. Execute with direct supervision and coaching
3. Execute with reactive supervision, i.e., on request, quickly available
4. Execute with indirect supervision, at a distance or post hoc
5. Execute without supervision
6. Supervise and train junior colleagues NYA Not Yet Assessable

| Task | Level of supervision | | | | | | |
| --- | --- | --- | --- | --- | --- | --- | --- |
|  | 1 | 2 | 3 | 4 | 5 | 6 | NYA |
| ❏ Explain ACR/SBI screening mammography guidelines and how they vary from USPSTF (starting age, interval, etc, why different recommendations, general statistics) |  |  |  |  |  |  |  |
| ❏ Correctly identify the 4 breast density parenchymal patterns |  |  |  |  |  |  |  |
| ❏ Differentiate technically adequate and inadequate studies |  |  |  |  |  |  |  |
| ❏ Differentiate benign findings from those that warrant additional work-up |  |  |  |  |  |  |  |
| ❏ Explain additional imaging needed in the diagnostic setting |  |  |  |  |  |  |  |
| ❏ Identify the normal and abnormal appearance of the breast after surgical procedures (reduction, augmentation, implants, breast conserving therapy, or mastectomy). |  |  |  |  |  |  |  |

| ❏ Demonstrate the correct use of the BI-RADS lexicon terminology pertinent to the examination including assessment/management categories. |  |  |  |  |  |  |  |
| --- | --- | --- | --- | --- | --- | --- | --- |
| ❏ Dictate concise report with few grammatical errors |  |  |  |  |  |  |  |
| ❏ Counsel patients and referring physicians about supplemental screening modalities (US, MRI) |  |  |  |  |  |  |  |
| ❏ Report and communicate results with the patient, referring physician |  |  |  |  |  |  |  |
| ❏ *Understand the basic requirements of the Mammography Quality Standards Act and Program (MQSA) as it pertains to screening mammography. |  |  |  |  |  |  |  |
| ❏ *Calculate basic screening mammography audit metrics including recall rate, positive predictive value 1 (PPV1), and cancer detection rate. |  |  |  |  |  |  |  |
| ❏ *Understand QA/QC requirements of analog and digital mammography. |  |  |  |  |  |  |  |

*These 3 assessments are better evaluated via knowledge test which is in the process of being created.

Comments:

**EPA 1 Teaching Tips: Identifying and managing abnormalities on screening examinations**

- **EPA1a: Mammography**
- **EPA1b: Ultrasound**
- **EPA1c: MRI**
- Have trainee work with technologist to understand mammogram positioning and technique
- Have trainee review screening cases they called back with you in person - have them discuss why they recalled and what diagnostic work-up they would perform.
- Discuss cases you called back that the trainee did not - explain why
- Have trainee discuss why a study is technically adequate/inadequate and how to correct images (mammogram, US, MRI)
- Have trainee correlate findings between screening ultrasound and mammography, or between screening mammography and diagnostic ultrasound
- Have trainee give precise history of why screening MRI was performed with pertinent positives and negatives

Once the trainee has interpreted 240-400 screening mammograms, 10-50 screening ultrasound (if applicable), 15-40 screening MRIs, and has performed 5-10 informal case-based discussions per modality under the supervision of an attending physician(s), they are ready to be evaluated on this EPA. If trainees have not met these numbers yet, they can still be evaluated on the EPA, with final score reflecting a data point used to demonstrate increased ability to perform tasks independently over the course of training.

*Each institution may have their own tracking system to calculate number of studies

A1.B. Radiation Oncology

The following is an example of an EPA developed for Radiation Oncology under the CBD initiative.^23,52^ The description of this EPA contains many of the sections described in Table 1, although its format differs. Similar to the previous example, their EPA description does not explicitly include “Potential risks related to failure modes.” It also does not include the “Frequency of practice required to maintain competence” section. The rest of the elements are included to various extents within the description, although in different order from what is listed in *Table 1.*

Copyright © 2025 The Royal College of Physicians and Surgeons of Canada. All rights reserved. Referenced and produced with permission.

For more information on the “Competence by Design” initiative:

<https://www.royalcollege.ca/en/standards-and-accreditation/competence-by-design.html>

**Radiation Oncology: Core EPA #3**

**Developing, evaluating, and implementing radiation treatment plans**

Key Features:

- This EPA integrates clinical factors, basic science principles and treatment planning logistics to develop, evaluate, and implement a radiation treatment plan, including selecting cases for peer review.
- This EPA also includes demonstration of basic technical proficiency in brachytherapy procedures.
- This EPA must be observed in a breadth of indications for radiation therapy, including case mix, case complexity, and treatment modality.
- The observation of this EPA is divided into three parts: plan development; plan evaluation; participating in brachytherapy procedures.

Assessment Plan:

Part A: Plan development

Direct or indirect observation by supervisor Use Form 1. Form collects information on:

- Case mix: breast; central nervous system (CNS) and eye; gastrointestinal (GI);

genitourinary (GU); gynecologic; head, neck and thyroid; lung/mediastinum; lymphoma/hematologic; skin; soft tissue/bone; pediatric oncology; benign condition

- Retreatment: no; yes
- Technique: cervix brachytherapy; prostate brachytherapy; IMRT; SRS; SBRT; IGRT; other

Collect 10 observations of achievement

- At least 6 different examples of the case mix
- At least 1 retreatment
- At least 1 cervix brachytherapy
- At least 1 prostate brachytherapy
- At least 1 SBRT
- At least 1 SRS
- At least 4 assessors

Part B: Plan evaluation

Direct observation by supervisor

Use Form 1. Form collects information on:

- Case mix: breast; central nervous system (CNS) and eye; gastrointestinal (GI); genitourinary (GU); gynecologic; head, neck and thyroid; lung/mediastinum; lymphoma/hematologic; skin; soft tissue/bone; pediatric oncology; benign condition
- Retreatment: no; yes
- Technique: cervix brachytherapy; prostate brachytherapy; IMRT; SRS; SBRT; IGRT; other
- Use of orthovoltage or electrons: yes; no

Collect 10 observations of achievement

- At least 6 different examples of the case mix
- At least 1 cervix brachytherapy
- At least 1 prostate brachytherapy
- At least 1 SBRT
- At least 1 SRS
- At least 1 retreatment
- At least 1 case using orthovoltage or electrons
- At least 4 assessors

Part C: Participation in brachytherapy procedures Direct observation by supervisor

Use Form 2. Form collects information on:

- Site for brachytherapy: cervical with tandem +/- ovoids; prostate (HDR or LDR); breast (HDR or LDR); vaginal vault; head and neck; gastrointestinal (esophageal, rectum, anal canal); lung; ocular plaque; other

Collect 2 observations of achievement

- At least 1 cervical brachytherapy
- At least 1 from any other brachytherapy treatment site

CanMEDS Milestones:

Part A: Plan development

1. **ME 3.5 Select the radiation prescription, providing rationale for dose/fractionation**
2. **ME 3.5 Use treatment simulation equipment effectively (including defining appropriate immobilization, patient preparation and techniques to minimize/account for organ motion)**
3. **ME 3.5 Integrate information from relevant clinical examinations and imaging (CT, PET, MR and Ultrasound), and pathology reports**
4. **ME 3.5 Perform contouring, applying the rationale for definition of GTV (+/- GTV2)**
5. **ME 3.5 Perform contouring, applying the rationale for definition of CTV (+/- CTV2)**
6. **ME 3.5 Perform contouring, applying the rationale for definition of PTV (+/- PTV2)**
7. **ME 3.5 Perform contouring of normal structures relevant to the case and prioritizing relative to targets**

Part B: Plan evaluation

1. **ME 3.5 Evaluate the radiation plan for target coverage**
2. **ME 3.5 Evaluate normal tissue constraints/dose to organs at risk**
3. **ME 3.5 Provide rationale for beam energy and dose modifiers**
4. **ME 3.5 Suggest modification or approve plan, as required**
5. **ME 3.5 Order appropriate verification imaging for treatment**
6. **L 1.1 Identify appropriate cases for oncology-related peer review and quality assurance activities**
7. **COM 5.1 Document radiation prescription plans in an accurate, complete, timely and accessible manner, and in compliance with legal and privacy requirements**

Part C: Participation in brachytherapy procedures

1. **ME 2.2 Interpret imaging studies to determine procedural plan**
2. **ME 3.4 Gather and/or manage the availability of appropriate instruments and materials**
3. **ME 3.4 Prepare and position the patient for the procedure**
4. **ME 3.4** Determine a plan for sedation and monitoring appropriate to the patient’s condition and the clinical setting
5. **ME 3.4 Apply knowledge of anatomy, key landmarks and the procedure**
6. **ME 3.4 Demonstrate aseptic technique where applicable: skin preparation, establishing and respecting the sterile field**
7. **ME 3.4 Participate in the procedure, including positioning the instrument accurately and as required**
8. **ME 3.4 Use image guidance where applicable including planning, registration, and navigation**
9. **ME 3.4** Determine that the procedure is complete
10. **ME 3.4** Monitor patient comfort and safety, and modify the procedure as needed
11. **COL 1.2 Communicate effectively with nurses and assistants during the procedure**
12. **ME 3.4 Identify and respond to immediate complications of the procedure**
13. **ME 4.1** Provide discharge instructions and plan for follow-up
14. **COM 5.1 Document the procedure**
